# A review of the off-label use of selamectin (Stronghold^®^/Revolution^®^) in dogs and cats

**DOI:** 10.1186/1751-0147-50-46

**Published:** 2008-11-25

**Authors:** Maggie A Fisher, David J Shanks

**Affiliations:** 1 Shernacre Enterprise, Shernacre Cottage, Lower Howsell Road, Malvern, Worcs WR14 1UX, UK; 2 Peuman, 16350 Vieux Ruffec, France

## Abstract

Since its introduction approximately seven years ago, selamectin (Stronghold^®^/Revolution^®^, Pfizer Inc.) has been used off-label to treat a number of ecto- and endoparasite conditions in dogs and cats. It has been used as a successful prophylactic against *Dirofilaria repens *and as a treatment for *Aelurostrongylus abstrusus *in cats. It has also been used to treat notoedric mange, infestation with the nasal mite *Pneumonyssoides caninum, Cheyletiella *spp. and *Neotrombicula autumnalis *infestations and larval *Cordylobia anthropophaga *infection. However, to date attempts to treat generalised canine demodicosis have not been successful. In all cases, treatment was apparently well tolerated by the host.

## Background

Until relatively recently, the antiparasitic products available to the veterinarian were often inadequate [1]. During the last two or three decades however, remarkable progress has been achieved in some areas of parasite control through better understanding of the behaviour and lifecycles of the target parasites and the introduction of a new generation of antiparasitics [1,2]. By the end of the last decade of the twentieth century avermectins (for example ivermectin and doramectin) and milbemycins (for example moxidectin), because of their activity against both endoparasites and ectoparasites, had become well established as endectocides for the treatment of livestock. The development of the equivalent products for cats and dogs evolved more slowly, perhaps because companion animals were not the priority for pharmaceutical development initially. The first avermectin-based product approved for use in companion animals was a low-dosage formulation of ivermectin solely for the prevention of adult heartworm (*Dirofilaria immitis*) infestations in dogs [3]. Higher doses of ivermectin, which might have provided a broader spectrum of activity allowing control of more parasite species, were unattainable because of idiosyncratic toxic reactions in some breeds of dog [4]. Thereafter a systematic programme to evaluate avermectin analogues resulted in the discovery of the macrocyclic lactone selamectin, which was shown to have efficacy and safety profiles which warranted its commercialisation for cats and dogs [5].

During the pre-development evaluation of the safety and efficacy of selamectin in dogs and cats, the compound was administered topically and orally at various intervals and dosages and its efficacy against target endo-and ectoparasites was assessed. In studies conducted early in the discovery and development process, selamectin was administered orally in some studies and topically in others. These studies demonstrated conclusively the efficacy of the compound, when applied topically, against both endoparasites (larval *Dirofilaria immitis*), and against ectoparasites (*Ctenocephalides felis*); oral administration showed activity against a range of parasites: *Ancylostoma caninum, A. tubaeforme, Toxocara canis, T. cati, Uncinaria stenocephala, Toxascaris leonina, Rhipicephalus sanguineus *and *Dermacentor variabilis *[5]. These early studies also confirmed the excellent safety profile of selamectin, even when administered orally to ivermectin-sensitive collies [5-7]. Further investigations demonstrated that topical application of a minimum of 6 mg selamectin per kg bodyweight gave prolonged efficacy against fleas [8] and the same dose-rate was effective in preventing the establishment of heartworm in dogs and cats [9]. After the successful completion of a global development programme, the topical formulation of selamectin was registered and is now marketed throughout the world (as either Stronghold^® ^or Revolution^®^, depending upon geographical region) as a topical product with a broad claims structure including a number of endo- and ectoparasites, for use on dogs and cats at a dose of at least 6 mg/kg (Table 1); it is commercialised in colour-coded tubes containing different amounts and different concentrations of the active ingredient, each suitable for animals in a defined weight band.

**Table 1:** Summary of label claims for selamectin in Europe

**Dogs and cats:** Treatment and prevention of flea (*Ctenocephalides *spp.) infestations including use as part of a treatment strategy for flea allergy dermatitis and may assist in the control of existing environmental flea infestations in areas to which the animal has access Treatment of ear mite (*Otodectes cynotis*) infection Heartworm (*Dirofilaria immitis*) prevention
**Dogs for the treatment of:** Adult *Toxocara canis* *Sarcoptes scabiei *(sarcoptic mange mites) *Trichodectes canis *(biting lice)
**Cats for the treatment of:** Adult *Toxocara cati* Adult hookworm (*Ancylostoma tubaeforme*) *Felicola subrostratus *(biting lice)

Although the minimum dose-rate of 6 mg/kg is identical for cats and dogs, there is evidence from pharmacokinetic studies that the topical treatment of cats resulted in approximately 50 times higher levels of selamectin within the animal compared to dogs [10]. This marked species difference is probably due to a series of factors including a greater flux through cats' skin than that of dogs and metabolic differences [10]. The higher bioavailability in cats may explain why selamectin appears effective against a broader range of endoparasites in cats than in dogs; for example, it is effective, when applied topically, against *A. tubaeforme*, the hookworm of cats, but not against *A. caninum*, the hookworm of dogs [5].

In the EU, veterinary surgeons can prescribe veterinary or even human medicines for dogs and cats to treat conditions where there is no specific label recommendation under the cascade system detailed in Directive 2001/82/EC, modified by 2004/28/EC. Such treatments are commonly referred to as "off-label" treatments. In common with most other veterinary products, there have been a number of published reports of the extra-label use of the commercialised topical formulation of selamectin against different parasites in the dog and cat; the purpose of this communication is to review these various reports and to discuss their implications for the current and future use of this important anti-parasitic product.

## Endoparasites

Endoparasite activity of selamectin is exerted against nematodes and it is in this area that the commercial product has been demonstrated to be effective.

There are believed to be five species of *Dirofilaria *worldwide and now that the control of *D. immitis*, the most important filarioid parasite of dogs and cats, is common practice in heartworm-endemic areas, attention is moving to the importance of other species of *Dirofilaria*. *D. repens*, which is endemic in southern areas of Europe, infects mainly dogs but can also infect cats. Adult worms are found most often in the subcutaneous connective tissue [11]. Infection may be asymptomatic, being discovered only when microfilariae are found in routine testing for *D. immitis *infection but infection has also been associated with pruritic dermatitis in some animals [11]. Moreover, as humans can also become infected, the parasite has important public health implications [12]. Genchi and co-workers [13] have recently reported that the commercial formulation of selamectin administered at monthly intervals according to label recommendations to prevent the establishment of *D. immitis *infections in dogs living in a *D. repens *endemic area and thus exposed to a high risk of infestation, was 100% successful in preventing the establishment of *D. repens *infection in 65 dogs; whilst 11 of 27 (41%) untreated controls in this study were shown to be infected following the transmission season, equating to an approximate incidence rate of 0.5116 per animal season at risk.

*Aelurostrongylus abstrusus *infects the lungs of cats and has a worldwide distribution; in areas where the infection is endemic up to 90% of cats may be infected [14,15]. Infection may be asymptomatic or may cause clinical signs, typically including severe coughing and respiratory distress. These are non-specific signs but diagnosis can be confirmed by examination of faeces or tracheal washings for infective larvae. In a case report [14], a six-month old domestic cat presented with a three-month history of dyspnoea, and aelurostrongylosis was diagnosed by the identification of first stage larvae in tracheal washings. The cat was treated with selamectin topically at 18 mg/kg and within two days clinical signs had receded and five weeks after the initiation of treatment (one week after a second application of selamectin at the same dose-rate) respiration was markedly improved and radiographic signs of bronchial disease were not evident. Selamectin at a dose-rate of 6 mg/kg was effective in one out of three cats on the basis of the elimination of larvae from the faeces after 30 days [16]. To date, reports of the use of selamectin to treat lungworm in dogs have not been published and it may be that selamectin is not useful in the treatment of lungworms in dogs, given the lower bioavailability of selamectin in dogs [10].

## Ectoparasites

Selamectin shows activity against both insect and arachnid classes of ectoparasites, and is licensed to treat sarcoptic mange and *Otodectes cynotis *infestations. As historically there has been a lack of effective miticidal treatments, and a greater lack of licensed miticidal treatments, unsurprisingly selamectin has been used against a number of mite infestations for which it does not have a label claim and has been demonstrated to possess useful activity against some species.

Notoedric mange is a highly contagious, pruritic cutaneous mite infestation of kittens, cats and occasionally rabbits, dogs and pine civets caused by the psoroptic mite *Notoedres cati *[17,18]. The infestation is clinically characterised by extreme pruritus and crusting lesions of the ears, head, neck, back and feet. *N. cati *is easily transmitted between animals and therefore a simple and reliable treatment must be rapidly applied to infested patients. A single administration of selamectin at the recommended dose (6 mg/kg) has been successfully used to eliminate the mites [17,18]. The success of a single treatment administered to cats in cases where eggs were evident at treatment suggests that selamectin has an extended duration of activity against *N. cati *in cats [17].

*Pneumonyssoides caninum*, the nasal mite of dogs, dwells in the caudal nasal cavity and paranasal sinuses of dogs causing non-specific signs of upper respiratory tract disease such as sneezing, reverse sneezing, epistaxis and impaired scenting ability [19]. The mite occurs worldwide but is particularly common in Scandinavia, where 20% of dogs in Sweden were found to be infected in a post-mortem survey [19]. Diagnosis in the living dog is difficult as the mites do not inhabit the easily visualised part of the nasal cavity, and in Scandinavia dogs with appropriate clinical signs are frequently treated presumptively. Gunnarsson and others [19] reported a controlled trial where six dogs infected with *P. caninum *were treated topically with selamectin on three occasions at fortnightly intervals at doses from 6 to 24 mg/kg. At post mortem examination between 33 and 35 days after treatment no mites were found in any treated dog, whilst five of six untreated controls had live mites.

One of the most striking clinical descriptions of an ectoparasite infestation of dogs, cats and rabbits is "walking dandruff", which may be observed on pups with a heavy infestation of *Cheyletiella *spp. This sign is caused by the movement of these rather large mites under "the bran-like exfoliative debris" [20] which occurs as a result of infestation [21]. Cheyletelliosis is highly contagious [21,22], particularly when a number of animals live together, as for example in breeding colonies [22] and is caused by *C. parasitivorax, C. blakei *or *C. yasguri*. *Cheyletiella *mites are not host specific [21,23], moving readily between dogs, cats and rabbits, and can survive for at least 10 days off the host in the environment under suitable conditions [21]. Infestation can occur by direct transmission of the parasite or via fomites, and the infestation is zoonotic [23]. Traditionally, infections were treated by weekly applications of various topical acaricidal products [21], with all the inconvenience and disturbance that such treatment regimens entail. Recently, selamectin administered topically has been demonstrated to be a highly effective treatment for cheyletiellosis in rabbits, cats and dogs [22]. Fifteen cats infested with *Cheyletiella *spp. and two uninfested dogs that lived in the same household were treated with between 6 and 15 mg, with a mean dose of 9 mg/kg selamectin/kg on days 0, 30 and 60 [21]. On day 120 no cat showed evidence of infestation and, during the follow-up of one year, re-infestation from environmental contamination or fomites did not occur, indicating that the infestation had been completely eliminated from the household. All 38 dogs in two households where persistent *Cheyletiella *sp. infestation had been identified were treated [22]. Dogs were treated with selamectin at a dose-rate of between 6 and 12 mg/kg at fortnightly intervals on a total of four occasions using the standard unit dosing tubes. Pruritus diminished following treatment and did not recur during a one year follow-up period. Although it cannot be proven that selamectin resulted in parasitological cure in these cases, administration at the stated dose rates appears able to resolve clinical signs of cheyletiellosis in dogs and cats.

Eight cats naturally infested with the harvest mite *Neotrombicula autumnalis *were treated with selamectin at 6 mg/kg and two days later the clinical signs associated with the infection had subsided and all mites were dead [24].

*Demodex *spp. are tiny, "cigar-shaped" mites which live in the hair follicles and sebaceous glands of mammals. Most infections are not associated with any clinical disease and it is not until much larger than normal populations infest the host, probably due to genetic characteristics or immunodeficiency of the host or to concomitant disease, that clinical manifestation of the infestation occurs [25]. In localised infestations on dogs, circumscribed areas of erythema and alopecia appear typically around eyes, mouth and on the forelegs; infestations which remain thus localised may self-cure [25] making it difficult to evaluate time efficacy of a product against localised infestations. However, the infestation may become persistent and generalised, with the hair becoming sparse over multiple areas of the body or feet, and in these cases the response to treatment may be poor. Attempts have been made to treat the generalised form of canine demodicosis with selamectin, applied both as recommended on the label and more frequently than recommended, applied to the recommended application site or directly to the visible lesions, but without success [26]. No improvement was seen in a Japanese Chin with generalised demodicosis which had previously proved unresponsive to ivermectin therapy when treated with selamectin once daily for two weeks at 30 mg/kg [27].

*Cordylobia anthropophaga *is found in Africa and is a myiasis-causing fly belonging to the family *Calliphoridae*. The adult *C. anthropophaga *or Tumbu fly lays eggs in the environment. When these larvae (the Cayor worm) hatch they remain in the environment until they sense a host, to which they rapidly attach prior to burrowing into the tissues. The larva develops subcutaneously with a single pore to the outside. It is a common cause of cutaneous myiasis in dogs in tropical Africa, including Senegal [28]. The clinical signs in the dog are erythematous nodular lesions of varying dimensions with a central pore from which there is a bloody discharge; the end of the parasite is visible through the pore. *C. anthropophaga *is of great importance in public health, since it also causes myiasis in man, and it is recognised that dogs and cats may carry the infection into previously unaffected areas. The efficacy of selamectin in the control of canine cordylobiosis was investigated in Dakar, Senegal [28] where 85% of dogs carried one or more larvae. Dogs were treated at the standard dose-rate of selamectin every 30 days. Treatment reduced the level of infestation to 0.1% by 10 days after the first treatment, and a similar low level of infestation was maintained throughout the remainder of the study, whilst 100% of dogs in the control group remained infested throughout the study.

## Discussion

Selamectin has been commercially available for approximately seven years and in that time has become established as a useful and effective treatment for those conditions for which it is licensed, and also as a readily-applied treatment for a range of other endo- and ectoparasites. As a spot-on treatment it may be useful for cats, for which it may be difficult for owners to apply or administer other treatments.

The picture of the relationship between pharmacokinetic characteristics and efficacy is still emerging. The most recent contribution [29] to the understanding of the pharmacokinetics of topical selamectin was a study with topical treatment administered at the minimum recommended dose-rate. The investigators found greater availability of selamectin in female beagles than in male following topical administration at a dose-rate of 6 mg/kg.

Treatment in all the cases described in this paper has been apparently well-tolerated, even in the Japanese Chin dog treated daily at 30 mg/kg (5 × the minimum recommended label dosage administered daily instead of monthly) for two weeks [27]. This is not unexpected as selamectin has a favourable safety profile [6,7], and is well-tolerated by cats and dogs including ivermectin-sensitive collies [5].

## Competing interests

The authors acknowledge the financial support of Pfizer Animal Health in conducting this review. Pfizer Animal Health provided the financial support to facilitate this review as a service to veterinarians in practice. Pfizer Animal Health does not endorse the use of selamectin other than in strict accordance with the product label.

DJS was an employee and consultant for Pfizer Animal Health for many years before retirement, and MAF has acted as consultant for Pfizer Animal Health on a number of projects.

## Authors' contributions

MAF and DJS participated in the drafting of the manuscript, in its revision for intellectual content and in the provision of references. Both authors read and approved the final manuscript.
